# Supplementary figures and images for: Optimizing Dosage-Specific Treatments in a Multi-Scale Model of a Tumor Growth
Source: Front Mol Biosci. 2022 Apr 6;9:836794. doi: 10.3389/fmolb.2022.836794 (PMC9019571; doi:10.3389/fmolb.2022.836794)

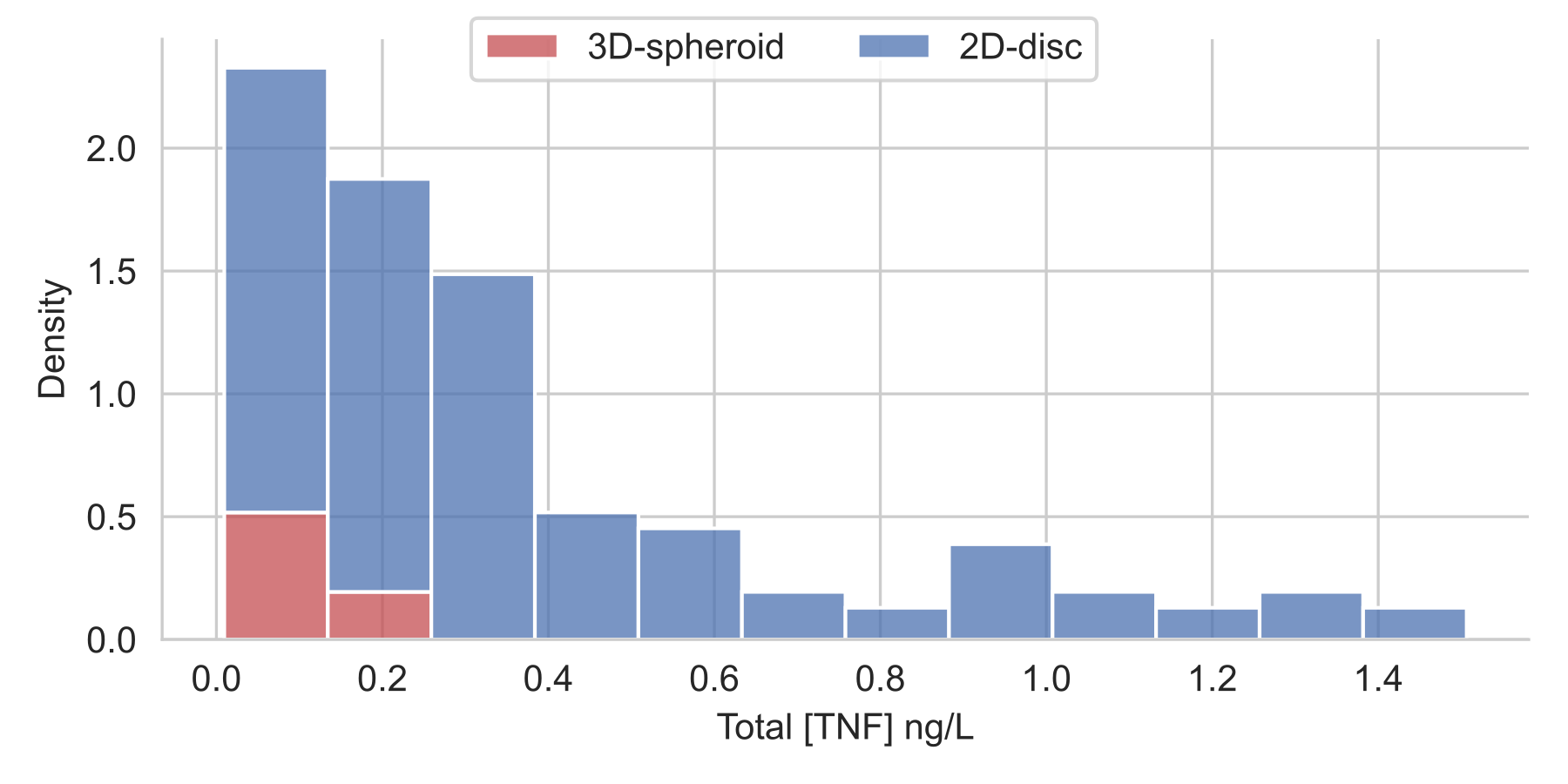

Supplement: Supplementary file 1 [file Image5.PNG]

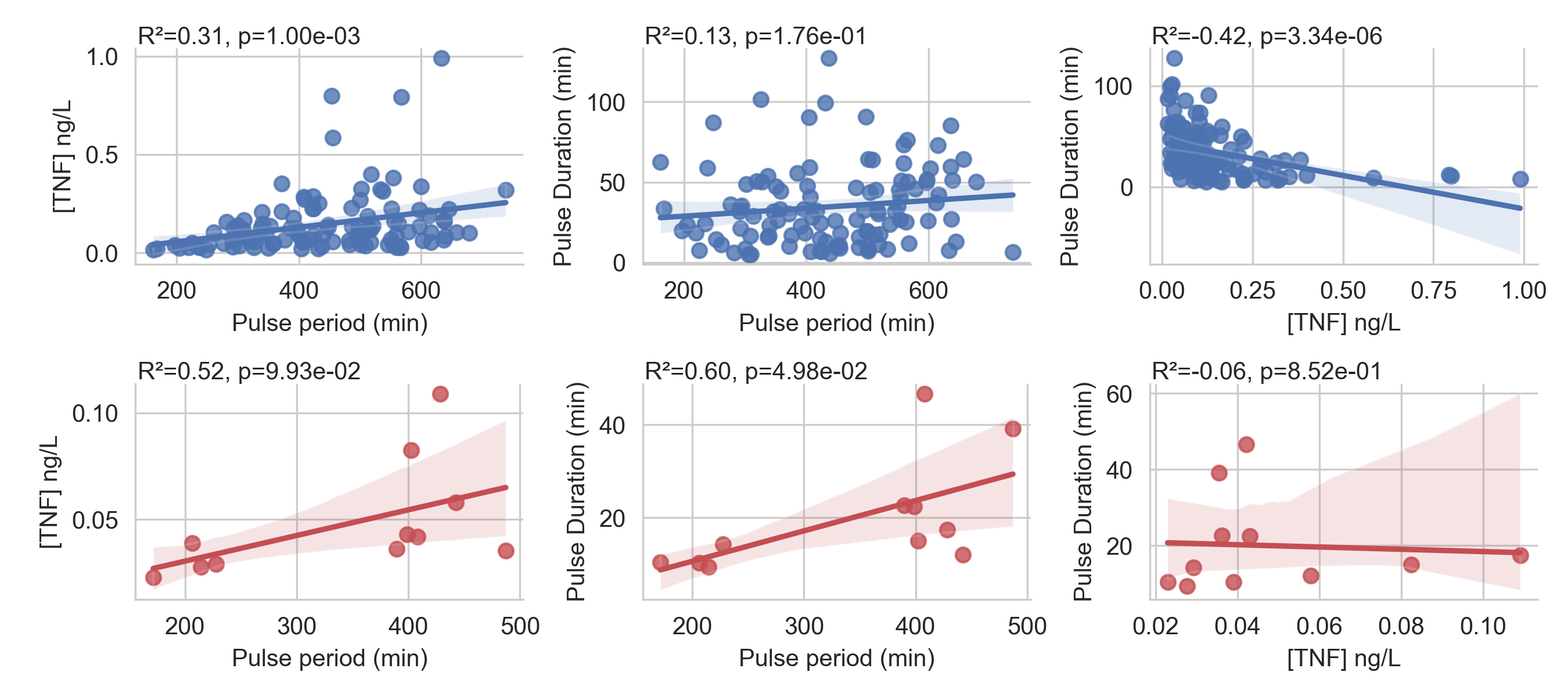

Supplement: Supplementary file 2 [file Image4.PNG]

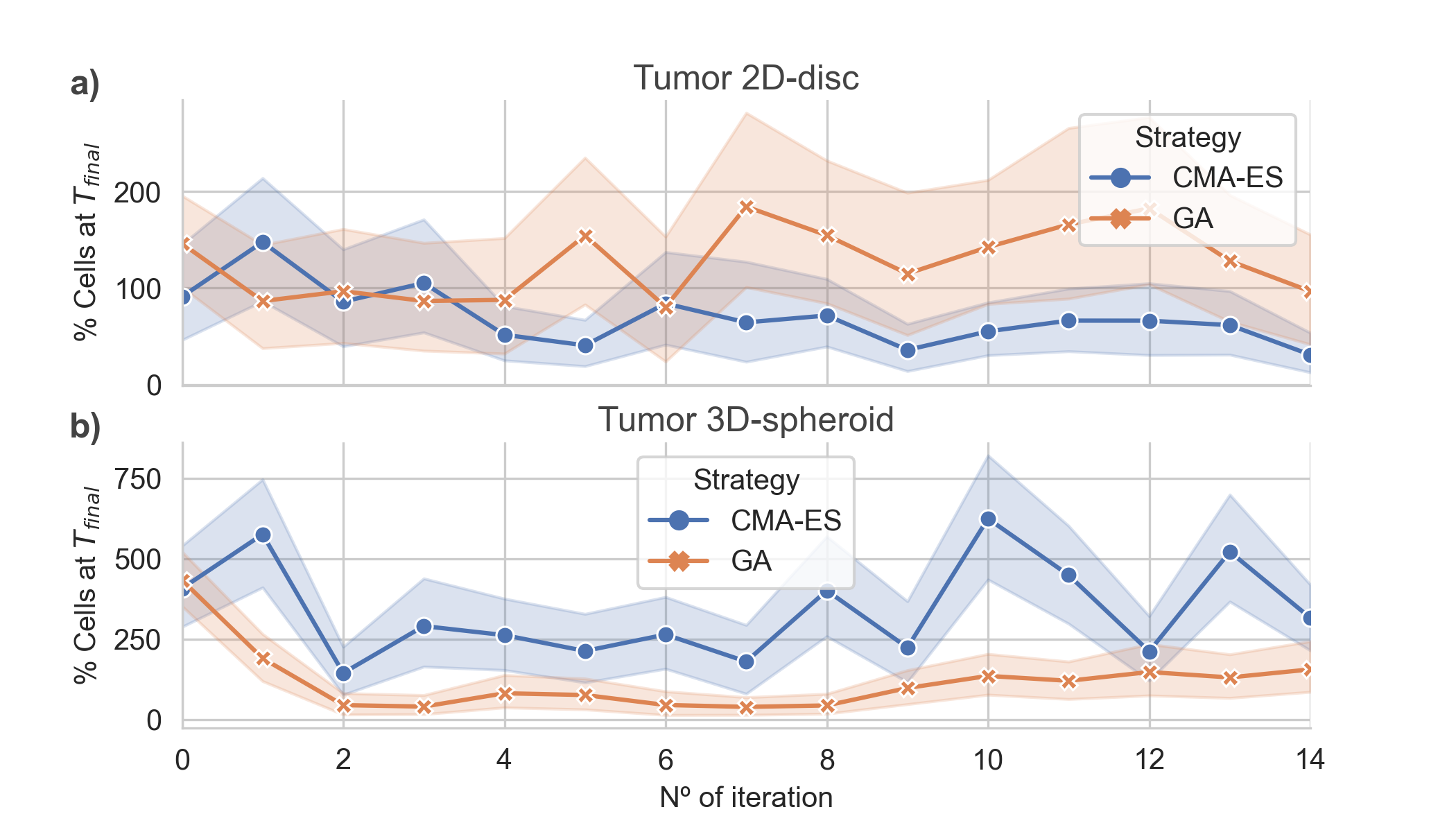

Supplement: Supplementary file 3 [file Image7.PNG]

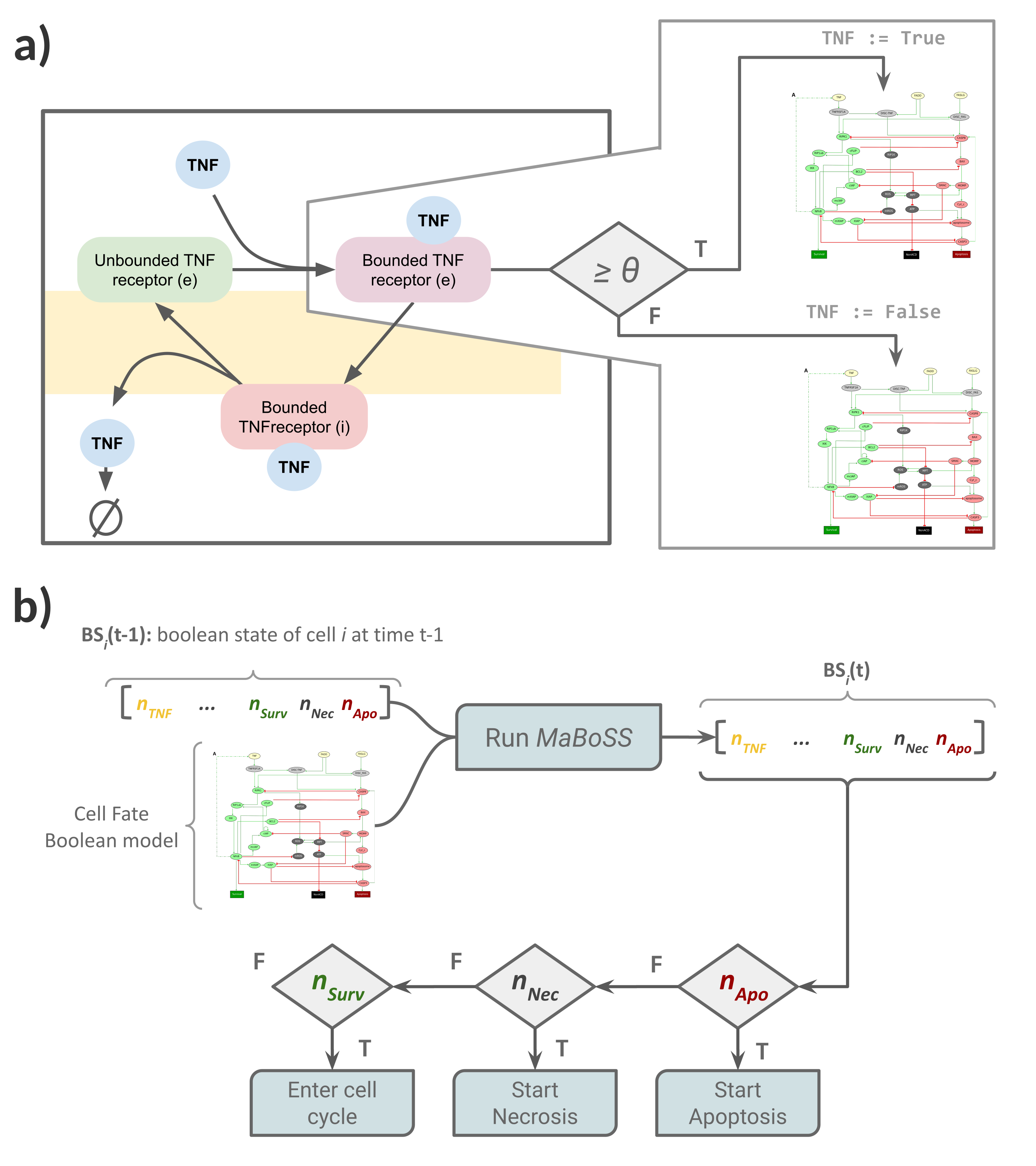

Supplement: Supplementary file 4 [file Image2.PNG]

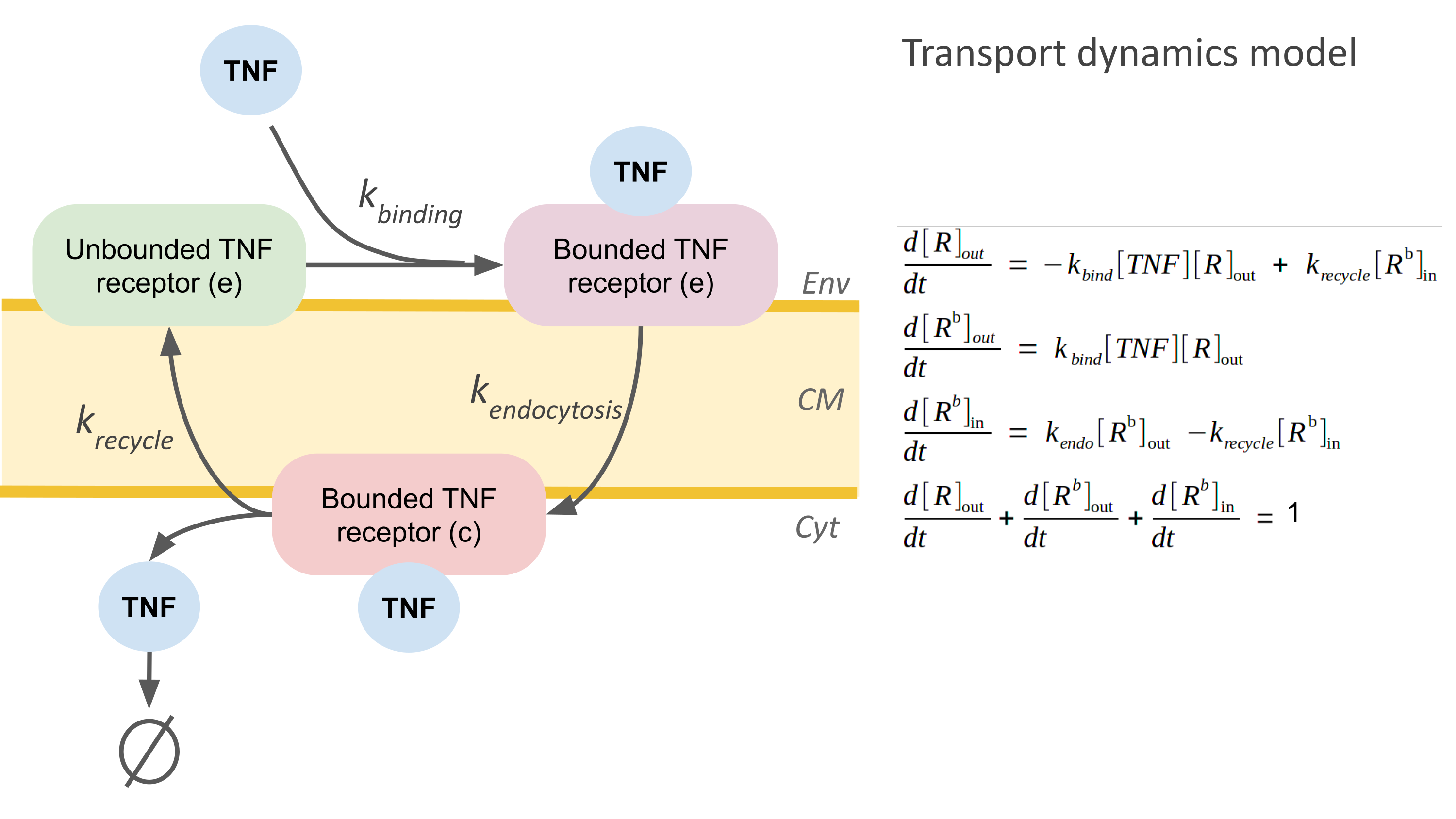

Supplement: Supplementary file 6 [file Image1.PNG]

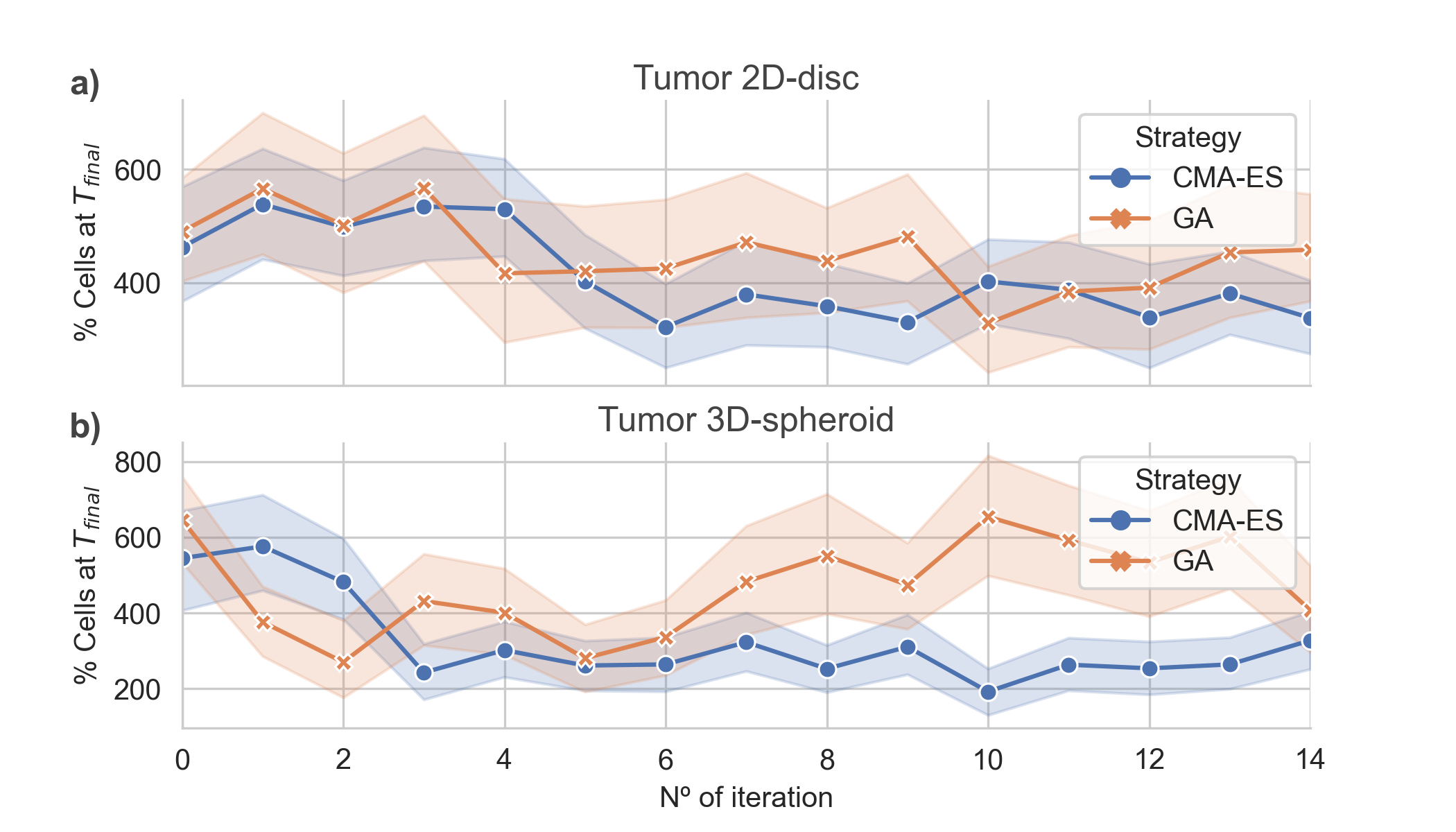

Supplement: Supplementary file 7 [file Image8.PNG]

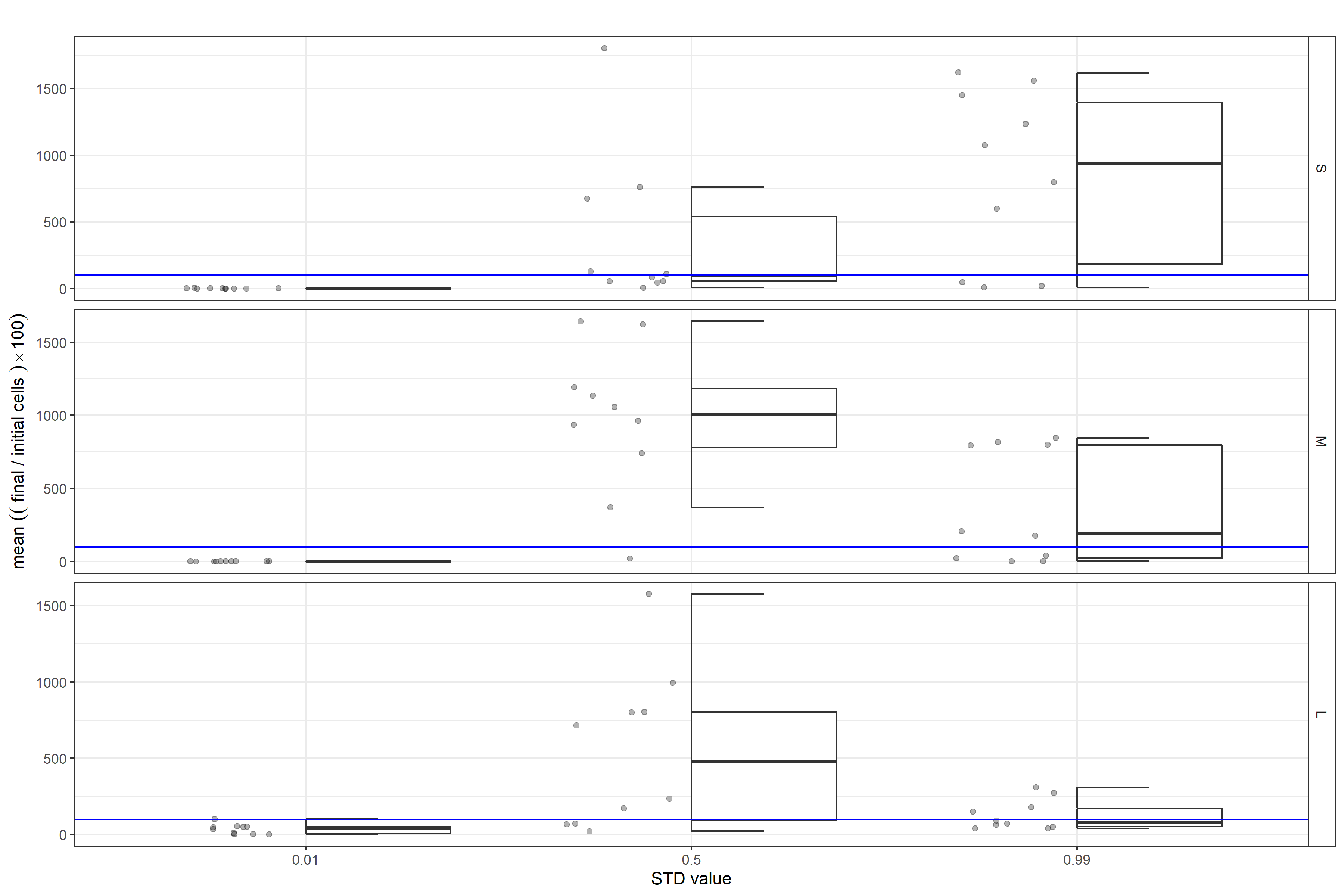

Supplement: Supplementary file 8 [file Image9.PNG]

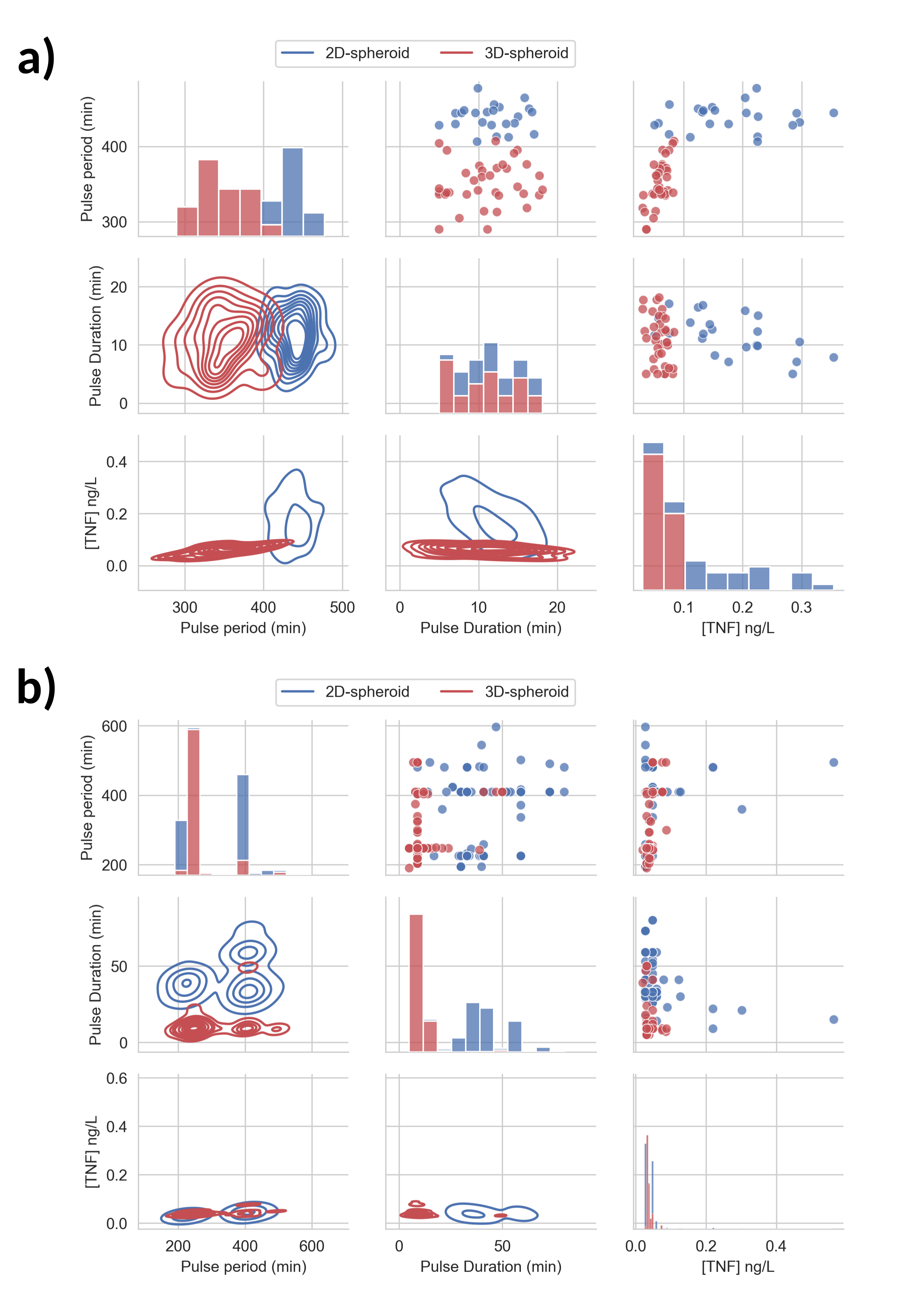

Supplement: Supplementary file 9 [file Image6.PNG]

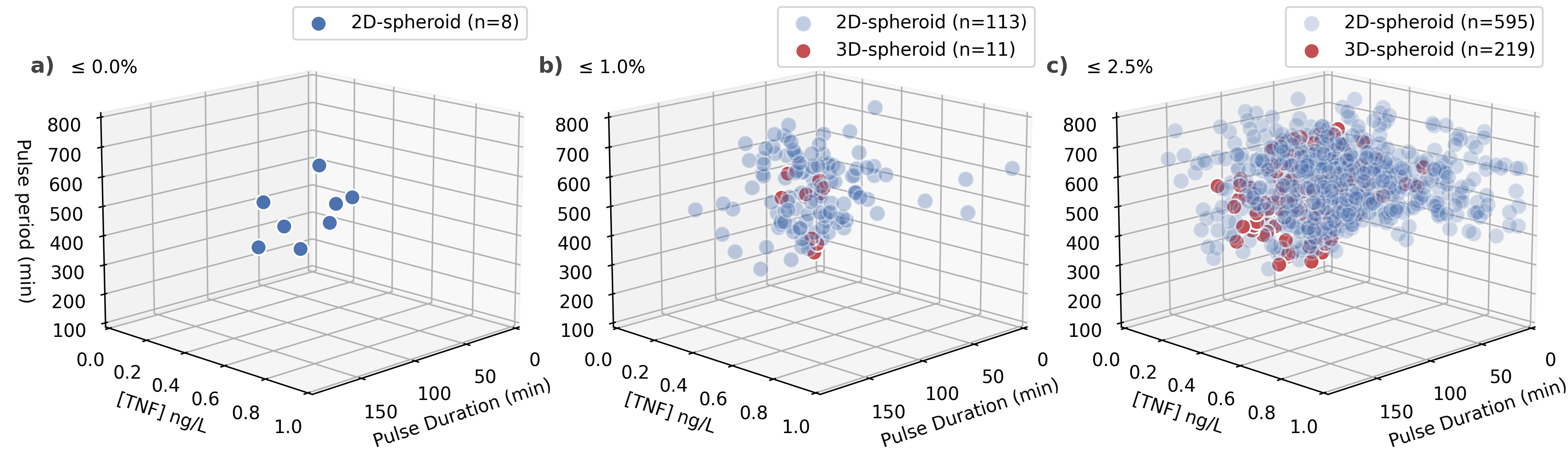

Supplement: Supplementary file 10 [file Image3.PNG]
